# Potential predictors of risk sexual behavior among private college students in Mekelle City, North Ethiopia

**DOI:** 10.11604/pamj.2017.28.151.5370

**Published:** 2017-10-18

**Authors:** Fanna Gebresllasie, Mache Tsadik, Eyoel Berhane

**Affiliations:** 1 Department of Ophthalmology, Quiha Hospital, Tigray, Ethiopia; 2 Department of Public Health, Mekelle University, College of Health Sciences, Mekelle, Ethiopia

**Keywords:** Risk sexual behavior, private college, multiple sexual partners, consistent condom use

## Abstract

**Introduction:**

Risk sexual practice among students from public universities/colleges is common in Ethiopia. However, little has been known about risk sexual behavior of students in private colleges where more students are potentially enrolled. Therefore, this study aimed to assess the magnitude of risky sexual behaviors and predictors among students of Private Colleges in Mekelle City.

**Methods:**

A mixed design of both quantitative and qualitative methods was used among 627 randomly selected students of private colleges from February to march 2013. Self administered questionnaire and focus group discussion was used to collect data. A thematic content analysis was used for the qualitative part. For the quantitative study, Univariate, Bivariate and multivariable analysis was made using SPSS version 16 statistical package and p value less than 0.05 was used as cut off point for a statistical significance.

**Results:**

Among the total 590 respondents, 151 (29.1%) have ever had sex. Among the sexually active students, 30.5% reported having had multiple sexual partners and consistent condom use was nearly 39%. In multivariable logistic regression analysis, variables such as sex, age group, sex last twelve months and condom use last twelve months was found significantly associated with risky sexual behavior. The findings of qualitative and quantitative study showed consistency in presence of risk factors.

**Conclusion:**

Finding of this study showed sexual risk behaviors is high among private colleges such as multiple sexual partners and substance use. So that colleges should emphasis on promoting healthy sexual and reproductive health programs.

## Introduction

Globally, young people aged 15-24 years are at the forefront of the epidemic and among the most vulnerable groups that account for an estimated 45% of new HIV infection [1]. In many developing countries adolescents have become increasingly prone to engage in risk sexual behaviors, because this age is characterized by a period of exploration and experimentation particularly in relation to sexual activity and they usually engage in risky sexual practices such as early sexual intercourse, multiple sexual partners, unprotected sexual intercourse, and non-regular partners such as commercial sex workers [2-5].

It is assumed that university and college students are fully aware of HIV risks and preventive mechanisms; how-ever, evidence showed that they are usually engaged in higher risk sexual behavior [6]. This risk sexual behavior accounts for a large number of opportunities for acquiring HIV infection. The number of sexual partners is an important indicator of risk sexual behavior. Many studies among sexually active University/college students indicated high rate of multiple sexual partners 40%, 10% and 6% in Mexico, Nigeria, and China respectively [5, 7, 8]. Similarly, a study conducted among University students of South Africa and Uganda also showed high rate of multiple sexual partners [9, 10]. Students in a higher years of study, non-resident students, students with frequent short and long distance mobility and students used alcohol reported multiple sexual partners [10].

Early age at first sex is associated with a long period of exposure to sexual activity, a higher tendency to have multiple sexual partners, and increased chances of acquiring sexually transmitted infections including HIV [11]. Despite this, the mean age at first sexual debut among University students was 17.7% [12]. In Ethiopia, a study conducted among Jimma university students showed 26.9% experienced sexual intercourse with males dominating about three times more likely to ever had sexual intercourse [12]. The only study in private college in Ethiopia also reported that 50.7% had sexual intercourse [13].

A survey study among Universities students in Ethiopia revealed a report of 33.5%, 28.3% and 50% as having multiple sexual partners respectively [12, 14, 15]. There is inconsistency in exposure to multiple partners among males and female students [12, 15]. Among those who experienced multiple sexual partners female, freshman students, Christian, single and alcohol users were more likely to engage in having multiple sexual partners [15].

Condom is considered as one of the main control strategies in the prevention of HIV and other sexually transmitted infections (STIs). Despite the expectation of University students to take preventive measures, the prevalence of consistent condom use was very low [14, 16-18]. Among the reasons for inconsistent condom use were being fresh man, alcohol, families residing in small cities and rural areas, having permanent relation, disliking using condoms and feeling that condoms decrease sexual satisfaction [15, 18].

A study showed that those who drink alcohol are less likely to use condoms consistently and more than a quarter of the sexually active students reported having sex under the influence of alcohol [8]. In fact alcohol use was associated with a doubled risk of having had an early sexual debut and risk of having had many sexual partners [10]. Peer pressure was also well recognized as main predisposing factors for unprotected sex and HIV infection [19].

The overall prevalence of HIV among community youth 15-24 is less than one. However, university and college students in the same age range are recognized as one of the most at risk population vulnerable to high risk sexual practice [11, 12]. Studies conducted in public Universities of Ethiopia showed potential exposure of students to risk sexual behavior [12, 14, 15, 18]. Despite this, there is scarce information regarding risk sexual behavior in private colleges where potentially high number of students gets enrolled in Ethiopia. Therefore, this study was aimed to assess the magnitude and predictors of sexual risk behavior among private college students.

## Methods

Institutional based cross sectional study was conducted using both quantitative and qualitative methods from February to March, 2013 in private colleges of Mekelle city. The study population comprised of regular students who were not married /in union and enrolled for 2012/2013 academic year in private colleges. The required sample size (n=627) was calculated using a single population proportion formula assuming that proportion of inconsistent condom use (55%) among Debrebrhan university students [17], 95% level of confidence, 5% margin of errors, design effect of 1.5 for the multi stage nature of the study and 10% adjustment was made for the non response.

$$n=\frac{{\left(Z\alpha /2\right)}^{2}.P(1-P)}{d^{2}}$$

Among the thirteen private colleges in Mekelle city, three colleges were selected randomly using a lottery method. Then the calculated sample size was distributed to each of the recruited colleges via proportional allocation to size. Again the allotted sample size was distributed proportionally based on the year of study. Finally, the study respondents were selected using simple random sampling from the prepared sampling frame or ID. For the qualitative part, student representatives and students who are participated indifferent clubs were selected purposively.

The quantitative part was collected using structured self-administered questionnaire. It was prepared in English, then translated to local language Tigrigna and then translated back to English and checked for consistency. The questionnaire had three components, information on socio demography, sexual behavior, and risk related factors. The questionnaires were pretested on 32 students in New Millennium College, Mekelle. Students who were selected to fill the questionnaire were assembled in separate rooms after obtained permission from the respected teachers and filled the questionnaire on similar day to avoid information contamination. For the qualitative method, four focus group discussions (2 male groups and 2 female groups) each with 8 FGD respondents who were not involved in the quantitative part were selected purposively. Besides, strict follow up and supervision was made by the principal investigator and supervisors during data collection. The collected data checked on site for completeness. Focus group discussion was facilitated and recorded by the principal investigator and supervisors, the raised issue was tape recorded.

In this study, risk sexual behavior is measured by two risk factors treated as outcome variables namely multiple sexual partner and condom inconsistency the last 12 months. The independent variables include socio-demographic characteristics, sexual behavior and related risk factors. Data entry was made using Epi-data version 3.1. Statistical analysis and cleaning was done using SPSS version 16. Descriptive statistics such as mean and standard deviation was used for continuous and frequency for categorical variables. Binary logistic regression was used to assess any significant association with the outcome variable. A separate multiple logistic regressions were made with each outcome variable. A confidence interval at 95% was used to compute the level of association and p-value at less than 0.05 was considered as a cutoff point for statistical significance. For the qualitative part, thematic analysis was made manually.

Ethical clearance and approval was obtained from the institutional review board (IRB) of Mekelle University. Permission was obtained from the respective colleges. Informed consent from students was taken prior data collection. Participation was voluntary and can withdraw at any time from the study. The collected data was kept confidential; no personal identifiers were recorded on the questionnaire. Moreover, the purpose, procedures of the study, advantages, and disadvantage were informed to respondents before the start of data collection.

## Results

### Socio economic and demographic characteristics

Out of the total 627 participants, 590 questionnaires were considered valid for analysis making the response rate of 96%. Of these respondents 428 (72.5%) were females. Nearly half of the study respondents293 (49.7%) were found in the age group of 15-19 years, and the mean age was 19.8 years with SD ± 2.1. Of the study participants, 91% were Orthodox Christians. Regarding their field of study nearly half of the study respondents were from non health fields (Table 1).

**Table 1:** Socio economic and demographic characteristics of students in private colleges of Mekelle city, Tigray, Ethiopia, 2013

Variable	Response	Male (162)	Female (428)
Age group	15-19	75 (25.6%)	218 (74.4%)
	20-24	87(29.3%)	210(70.7%)
Religion	Orthodox	147 (27.4%)	390 (72.6%)
	Muslim	12 (33.3%)	24 (66.7%)
	Others	3 (17.6%)	14 (82.4%)
Year of study	I Year	87 (38.8%)	137 (61.2%)
	II Year	49 (22.4%)	170 (77.6%)
	III Year	26 (17.7%)	121 (82.3%)
Previous Residence	In the city	82 (24.3%)	255 (75.7%)
	Outside the city	80 (31.6%)	173 (68.4%)
With whom do you live now	Parents	89 (24.0%)	282 (76.0%)
	Relatives	3 (13.0%)	20 (87.0%)
	In rent house	65 (35.7%)	117 (64.3%)
	Boy /girl friend	5 (35.7%)	9 (64.3%)
Pocket money	Yes	85 (30.0%)	198 (70%)
	No	77 (25.1%)	230 (74.9%)

### Sexual and other related behavior of respondents

Out of the total 590 respondents 25.6% have ever had sex with someone. Of these sexually active respondents 53% were males. The mean age at first sexual intercourse was 18.3 years with standard deviation ± 2. The main reason cited by respondents for first sex was sexual desire 55.6%. Out of the total sexually active students, 79.5% reported for having sex with their boy or girl friend and the rest reported sex with commercial sex workers. Some of the risk behaviors reported by the respondents were multiple sexual partners (30.5%), substance use such as alcohol and Khat (27.1%) and watching pornographic movies (39.8%) (Table 2).

**Table 2:** Sexual and other related behaviors among private college students of Mekelle city, Tigray, Ethiopia, 2013

Variable	Response	Male	Female
Ever had sex (n=151)	Yes	80 (53%)	71 (47%)
	No	82 (18.7%)	357 (81.3%)
Age at first sex	< 18 years	60(75%)	20(25%)
	>18 years	30(42.3%)	41(57.7%)
Condom use at first sex	Yes	57(64.8%)	31(35.2%)
	No	23(36.5%)	40(63.5%)
condom consistency	Yes	36(70.6%)	15(29.4%)
	No	44(44%)	56(56%)
Sex last 12 month	Yes	60 (51.3%)	57 (48.7%)
	No	20(58.8%)	14(41.2%)
Multiple sexual partner last 12 months	Yes	28(77.8%)	8(22.2%)
	No	32(39%)	50(61%)
Substance use	Yes	79(49.4%)	81(50.6%)
	No	83(19.3%)	347(80.7%)
Type of substance	Alcohol	55(52.9%)	49(47.1%)
	Khat/Hashish	24(42.9%)	32(57.1%)
Close friends encourage to use substance	Yes	45(61.6%)	28(38.4%)
	No	32(47.8%)	35(52.2%)
Close friends encourage sexual intercourse	Yes	64(59.8%)	43(40.2%)
	No	38(35.5%)	69(64.5%)
Watching pornography	Yes	107(45.5%)	128(54.5%)
	No	55(15.5%)	300(84.5%)

### Inconsistent condom use

In bivariate regression, variables such as sex, residence before joining college, had sex in the last 12 months, watching pornographic movies and having friends had sexual experience were found significant associated with inconsistent condom use. However, in multivariate regression only sex and had sexual intercourse in the last 12 months were found significantly associated. Female students were more likely to use condom inconsistently compared to male counterparts [AOR = 2.47, (95% CI: 1.05, 5.83)] (Table 3).

**Table 3:** Factors associated with inconsistent condom use among private college students of Mekelle city, Tigray, Ethiopia, 2013

Variables	Response	Inconsistent condom use		COR	AOR
		Yes	No		
Sex	Male	44	36	1.00	1.00
	Female	56	15	3.05(1.48,6.27)^+^	2.47(1.05, 5.83)^+^
Age group	15-19	30	15	1.00	
	20-24	70	36	1.030(0.49,2.15)	
Previous residence	In the city	21	61	0.45(0.22,0.89)^+^	0.49(0.22,1.06)
	Outside the city	30	39	1.00	1.00
Sex last 12 months	Yes	47	70	5.04(1.66,15.23)^+^	6.03(1.87,19.42)^+^
	No	4	30	1.00	1.00
Substance use	Yes	87	46	1.37(0.46,4.09)	
	No	13	5	1.00	
Watch pornographic film	Yes	39	59	2.25(1.06, 4.83)^+^	1.43(0.53,3.80)
	No	12	41	1.00	1.00
Close friends with sexual experience	None of them	10	34	1.00	1.00
	Few of them	35	63	1.89(0.83,4.27)	1.36(0.52,3.57)
	Many of them	6	3	6.80(1.44,32.19)^+^	3.92(0.68, 22.35)

+ P-value < 0.05

### Reasons for non use of condom

The main reasons mentioned by sexually active students for never using condom were not comfortable 41.3%, not accessible 19%, believing of their partner 17%, religious reason 10% and embarrassed to buy condom 8%. More over sexually inactive students were asked the reasons for not having sex with someone, 247 (51.9%) reported to wait until marriage, of these 220 (89.1%) were females and 27 (10.9%) were males.

### Multiple sexual partners

In bivariate logistic regression variables such as sex, age group, condom last twelve months, watching pornographic films, close friends currently using substance and having close friends started sexual intercourse were found statistically significant. These variables were entered to multivariable logistic regressions. After adjustment for potential confounding variables age group, sex and condom use last twelve month were showed significant association with having multiple sexual partners. The finding indicated that, the odds of having multiple sexual partners were nearly five times higher among the age group of 20-24 years compared to those 15-19 years [AOR=4.82(95%5 CI: 1.52, 15.31)]. Male students were more likely engaged to multiple sexual partners compared to female students [AOR = 4.33, (95% CI: 1.44, 12.99)]. In the other hand, the likely hood of having multiple sexual partners was higher among students using condom in the last 12 months compared to non users (Table 4).

**Table 4:** Factors associated with multiple sexual partners among private college students in Mekelle city, Tigray, Ethiopia, 2013

Variables	Response	Multiple sexual partner		COR	AOR
		Yes	No		
Sex	Male	28	32	5.47(2.23,13.48)^+^	4.33(1.44, 12.99)^+^
	Female	8	50	1.00	1.00
Age group	15-19	6	27	1.00	1.00
	20-24	30	55	2.45(1.91, 6.61)^+^	4.82(1.52, 15.31)^+^
Year of study	Year I	27	13	1.00	
	Year II	32	15	1.02(0.49,3.92)	
	Year III	23	8	1.16(0.49,3.71)	
Previous residence	In the city	19	44	0.89(0.39, 2.02)	
	Out of city	15	31	1.00	
Condom use last 12 months	Yes	32	49	5.39(1.74,16.66)^+^	3.48(1.01, 12.00)^+^
	No	4	33	1.00	1.00
Sex for money	Yes	3	3	2.29(0.24,9.57)	
	No	34	78	1.00	
No of friends who had sexual experience	Never of them	6	29	1.00	1.00
	Few of them	24	51	2.27(0.83,6.21)	1.35(0.38, 4.77)
	Many of them	6	2	14.50(2.34, 90.02)^+^	3.87(0.43,31.89)
Friends use substance	Yes	22	29	1.00	1.00
	No	14	53	2.87(1.28, 6.45)^+^	1.62(0.56,4.70)
Watching pornography	Yes	30	47	1.00	1.00
	No	6	35	3.87(1.39, 9.92)^+^	1.47(0.40,5.38)

+ P value < 0.05

### Summary of qualitative data to supplement the gap

The focus group discussion (FGD) was conducted among 2 female and 2 male groups each with 8 discussants. Majority of discussants were in the age range of 18-24 years. The average time taken for each group was 1:00-1:15 h. The discussants were from all the selected private colleges for the study where the quantitative study was conducted.

Discussants were asked to express the sexual behavior of students in their respective college. A female discussant replied that some students engage in sexual practice to get financial and educational support from others regardless of the risk involved. A male discussant revealed that a number of students live in rented houses and lead independent life without the control of families which predispose them to risky sexual behavior.

A premarital sexual practice is also common among private college students and more female students have a boyfriend and practice sex. Many students perceived that school life without a boyfriend or girlfriend is considered as “Fara” meaning know nothing as discussants mentioned. The effect of peers and longtime acquaintance expose students to practice unsafe sex.

Regarding safer sex practice, respondents responded that “majority of the college students are practicing safe sex by using condom and emergency pills. However, some students placed themselves on risky sexual behavior such as having two or more sexual partners and inconsistent condom use despite their awareness of HIV/AIDS. Discussants noted that some students considered HIV/AIDS like a “common cold” and some students went to night clubs every weekend and drink pocket or bag. Use of substances like alcohol, hashish and shisha was also reported as commonly used by college students particularly at night clubs. Female students often use shisha because they believe that it has the effect of “shining the face”. As the discussants said Khat also causes post exam sleep disturbance, then students took alcohol to overcome the problem and engaged in unsafe sex.

Majority of respondents mentioned HIV, unwanted pregnancy, health problems and addiction as outcomes of risky sexual behavior. Respondents also declared that colleges lack focus in counseling sexual and reproductive health issues. Lastly, the discussants suggested the need for education and counseling programmes to create awareness to private college students.

Majority of respondents mentioned HIV, unwanted pregnancy, health problems and addiction as outcomes of risk sexual behavior. Respondents also declared that colleges lack focus in counseling sexual and reproductive health issues. Lastly, discussants suggested the need for education and counseling of risk behavior to create awareness to private college students.

## Discussion

This study aimed to assess the magnitude of risk sexual behavior and associated factors among private colleges in Mekelle city. The finding of this study showed the mean age and median age for sexual initiation was 18.3 ±2 and 18 years respectively, which is similar with findings from private colleges in Bahrdar city (18.7±1.9), and study conducted in Jimma University (17.7±2.7) [12,13]. However, the median age was slightly lower than studies conducted among female students of South Africa; the median age at first sexual intercourse was 19.0 years [9].

Among the study participants, nearly 26% had experienced sexual intercourse which is more consistent with the research findings of Jimma University (26.9%) [12]. But, in a study conducted in Durban South Africa more than half of the students (58.8%) reported presently being sexually active [9]. In line with this study done in Bahrdar private college students showed, ever had sexual intercourse was reported by (50.7%) which is much higher than this study [13]. The reason for this high disparity could be difference in college based risk education and information provision to students. In addition there may be also social and cultural differences as well as the difference in the settings of the cities such as possessing recreational areas.

The magnitude of multiple sexual partners in this study was 30.5 % which is much lower than studies conducted in Haramaya University (33.5%) [14] and Hosanna health science colleges (47.6%) [15]. However, it was found higher than the study conducted in Jimma University (28.3%) [12]. Having multiple sexual partners was also reported as a common practice among youth in a study conducted in Addis Ababa [20]. According to the FGD report, having multiple sexual partners is a common practice which supports the findings from the quantitative study

Sex was a predictor for having multiple sexual partners in this study. It indicates that among those sexually active participants, the odds of having multiple sexual partner is more than four times higher among males compared to females which is consistent with the finding of Bahrdar Private college [13]. Another study conducted in Durban, South Africa showed that sexually active male students had higher rate of multiple partners compared to female students (49.8% vs 9.8%) [9]. The possible reason for males to have multiple sexual partners could be the accepted norm of males to have more than one partners. In contrast, a result of a study conducted in Hosanna health science college showed that females had more likely to have multiple sexual partners [15]. The reason for having multiple sexual partners among females in the specified study area might be due to diversity in the socio-cultural factors.

In this study, student’s age was found significant for having multiple sexual partners; those in the age group of 20-24 years were found nearly five times more likely to have multiple sexual partners when compared to their younger age group. This could be because of age related sexual experience and their relative skill to communicate with others for sexual act.

Regarding consistency of condom use, in this study 66.2 % of respondents use condom inconsistently which is incomparably lower than the study conducted in Haramaya University (79.6%) [14]. This might be justified as substance use in Haramaya is a common practice and this may predispose students to use condom inconsistently. Other study conducted in Hosanna College showed, only 20 % of respondents were inconsistent condom users [15] which showed a higher disparity compared with this study finding. The possible explanation for the disparity may be a difference in socio-cultural and behavioral factors. The finding of this study showed inconsistent condom use was higher among female students compared to their male counter parts. This is consistent with the report of FGD respondents putting their remark as many students went to alcohol houses and night clubs which make them to lose their ability to think and puts them on danger of unprotected sex, though they have condom in their hand.

In this study, sex in the last 12 months was reported by 77.5 % of students which is much higher than studies conducted in Haramaya University (50.9%) and Jimma University (51%) [12,14]. The reason for the higher prevalence in this study may be, because most of the students from private colleges have a little bit different living style and they leave out of campus and out of parent’s control in rented house which exposed them for unsafe sexual practice. Moreover, the qualitative part (FGD) of this study indicated that having a sexual partner is a usual phenomenon and considered as a sign of modernalization.

## Conclusion

A considerable proportion of students in private colleges practice risk sexual behavior which endangers their future. Accordingly, 66.2% had reported condom inconsistency and 30.5% had multiple sexual partners. Among the predictors, variables such as sex, age group, sex in the last 12 months and condom use last 12 months were found statistically significant with condom inconsistency and multiple sexual partner which is supported by the finding from qualitative part. This implies the need for concerted effort to alleviate the problem. Therefore, private college communities need to focus on promotion of healthy sexual and reproductive health issues through counseling and education programmes.

### What is already know on this topic?

- The magnitude of risk sexual behavior among public university/colleges students is high in Ethiopia;
- Risk behaviors such as multiple sexual partner and inconsistent condom use among public university/college students is significantly high.

### What this study adds

- When the practice of risk sexual behavior among private college students is qualitatively explored , students perceived that having sex is a sign of modernization;
- College students are more vulnerable to risk sexual behaviors than public university/college students because of the different living style and exposure (live in rent house in groups which exposes to peer effect).

## Competing interests

The authors declare no competing interests.
